# A Transient Pulse of Genetic Admixture from the Crusaders in the Near East Identified from Ancient Genome Sequences

**DOI:** 10.1016/j.ajhg.2019.03.015

**Published:** 2019-04-18

**Authors:** Marc Haber, Claude Doumet-Serhal, Christiana L. Scheib, Yali Xue, Richard Mikulski, Rui Martiniano, Bettina Fischer-Genz, Holger Schutkowski, Toomas Kivisild, Chris Tyler-Smith

**Affiliations:** 1 Wellcome Sanger Institute, Wellcome Genome Campus, Hinxton CB10 1SA, UK; 2 The Sidon excavation, Saida, Lebanon; 3 Department of Archaeology and Anthropology, University of Cambridge, Cambridge CB2 1QH, UK; 4 Department of Archaeology, Anthropology, and Forensic Science, Bournemouth University, Talbot Campus, Poole BH12 5BB, UK; 5 Department of Genetics, University of Cambridge, Downing Street, Cambridge CB2 3EH, UK; 6 Publication Department, Orient-Institut Beirut, Beirut 11-2988, Lebanon

**Keywords:** aDNA, medieval period, Roman period, whole-genome sequences, Lebanon, Sidon, population history

## Abstract

During the medieval period, hundreds of thousands of Europeans migrated to the Near East to take part in the Crusades, and many of them settled in the newly established Christian states along the Eastern Mediterranean coast. Here, we present a genetic snapshot of these events and their aftermath by sequencing the whole genomes of 13 individuals who lived in what is today known as Lebanon between the 3^rd^ and 13^th^ centuries CE. These include nine individuals from the “Crusaders’ pit” in Sidon, a mass burial in South Lebanon identified from the archaeology as the grave of Crusaders killed during a battle in the 13^th^ century CE. We show that all of the Crusaders’ pit individuals were males; some were Western Europeans from diverse origins, some were locals (genetically indistinguishable from present-day Lebanese), and two individuals were a mixture of European and Near Eastern ancestries, providing direct evidence that the Crusaders admixed with the local population. However, these mixtures appear to have had limited genetic consequences since signals of admixture with Europeans are not significant in any Lebanese group today—in particular, Lebanese Christians are today genetically similar to local people who lived during the Roman period which preceded the Crusades by more than four centuries.

## Main Text

Human migrations, which often accompanied historical battles and invasions, have profoundly reshaped the genetic diversity of local populations in many regions. The Mongols, around Genghis Khan’s time, spread their male lineages throughout Asia from the Pacific to the Caspian Sea,^1^ while in South America, the arrival of colonial Iberians resulted in the European ancestry becoming the major component in the genomes of many South Americans today.^2^ But do all mass migrations leave genetic imprints in local populations? For two centuries, starting in 1095 CE, hundreds of thousands of Europeans arrived in the Near East to fight in the Crusades and to settle in the newly established European states along the Eastern Mediterranean coast.^3^ The historical records from this period report varying episodes of forced displacement of the local people, or co-existence and mixing with them.^3^, ^4^ We have previously reported finding European Y chromosome lineages in the present-day population of Lebanon^5^ and suggested those could have originated from the Crusaders when Lebanon was under their rule during the medieval period. However, more recently, using whole-genome sequences from modern and ancient individuals, we found that present-day Lebanese derive most of their genetic ancestry from the local Bronze Age population and from a Eurasian Steppe-related admixture which occurred around 1,750–170 BCE.^6^ Thus, the Lebanese autosomal genomes appear not to have been impacted by the Crusades.

Ancient DNA (aDNA) from medieval Crusaders who traveled to the Near East and from local people who were their contemporaries and interacted with them can potentially resolve the discrepancy between the historical records reporting admixed descendants of Crusaders in the Near East and the genetics of modern populations not displaying such a signal. aDNA from this period can also resolve uncertainties in the demographic processes that accompanied the Crusades, providing insight into questions such as what was the provenance of the Crusaders’ armies and the extent to which they were from genetically and geographically diverse groups. How genetically similar are modern Near Easterners to the medieval populations and what genetic changes occurred after the Crusades? These questions can potentially be answered today in great detail using ancient genomics; however, obtaining aDNA from Crusaders is challenging and hindered by two major factors: the hot and humid climate in the Near East negatively impacts the survival of aDNA^7^ and burials of individuals who can be linked to the Crusaders are rare.^8^ One of the few known Crusader burial sites is located in the city of Sidon (in the south of present-day Lebanon), an important stronghold in the Crusaders’ Kingdom of Jerusalem and a scene of major battles between the Crusaders and the Arabs from 1110 CE to 1249 CE (Figure S1). A recent archaeological excavation in Sidon near the ruins of a Crusader castle uncovered two mass burials consisting of skeletal remains from a minimum of 25 individuals who had signs of inter-personal violent injuries and dated using radiocarbon to 1025–1283 calCE (calibrated date) (Table S1). The bodies were placed in arbitrary manner into two adjacent simple pits dug in the ground (Figure S2). The location, date, and condition of the burials, together with a Crusader coin issued in Italy in 1245–1250 CE^9^ and five buckles with designs associated with medieval Europe,^8^ all suggest that the burials could have been for Crusaders killed in battle during the 13^th^ century CE.

We sampled the petrous portion of the temporal bones from 16 of these individuals and, additionally, sampled five local individuals from an archaeological excavation in Qornet ed-Deir, Jabal Moussa UNESCO Biosphere Reserve in Mount Lebanon (Figure S3),^10^ who lived during the Roman period (237–632 calCE), and thus these would represent the local ancestry before the time of the Crusades. We extracted DNA, built double-stranded libraries according to published protocols,^11^, ^12^, ^13^ and sequenced the libraries on an Illumina HiSeq 2500 using 2 × 75 bp reads. We processed the sequences using PALEOMIX,^14^ retained reads ≥30 bp, and collapsed pairs with minimum overlap of 15 bp and a mismatch rate ≤0.1. We mapped the merged sequences to the *hs37d5* reference sequence, removed duplicates, removed bases from the end of the reads until the frequency of nucleotide misincorporation dropped to below 5%, and randomly sampled a single sequence with a minimum base quality of ≥20 to represent each SNP. We found that seven samples from the Crusaders’ pit had less than 2% of endogenous DNA from the first exploratory sequencing runs and thus we excluded them from additional sequencing and from further analyses. The remaining nine samples from the Crusaders’ pit and all five samples from the Roman period had 2% to 58% endogenous DNA with post-mortem damage patterns typical of ancient DNA (Figure S4) and produced genomic coverage between 0.4× and 4.4×, plus mitochondrial DNA (mtDNA) genome coverage between 29× and 415× (Table 1). We estimated contamination from the mtDNA genome of all individuals and the X chromosomes of males^15^, ^16^ and found that the sequence data were minimally contaminated (Table S2), except for sample QED-9 (from the Roman period) who had 8%–10% estimated mtDNA contamination; thus this individual was not included in subsequent analyses.

**Table 1:** Samples Analyzed in This Study

**ENA Number**	**ID**	**Excavation Site**	**Period**	**Date**^a^**(calCE)**	**Mapped Reads**	**Mapped Read %**	**Coverage Genomic**	**Coverage Mt**	**Sex**^b^	**Mt Haplogroup**	**Y Haplogroup**
ERS3189349	SI-38	Sidon	medieval	–	33711271	4	0.5	72	male	J1b4a1	E-L677
ERS3189350	SI-39	Sidon	medieval	1191–1283	35804710	3	0.6	60	male	H5′36	R-P312
ERS3189351	SI-40	Sidon	medieval	–	21871263	2	0.4	38	male	U5a1g	R-P311
ERS3189352	SI-41	Sidon	medieval	1187–1266	169086445	11	3	198	male	HV0a	R-DF27
ERS3189353	SI-42	Sidon	medieval	1154–1281	37079898	5	0.7	68	male	J1b1a1	T-M70
ERS3189348	SI-44	Sidon	medieval	–	25262493	3	0.4	52	male	HV1b	J-M304
ERS3189355	SI-45	Sidon	medieval	1219–1278	193449685	12	3.75	275	male	J1d1a1	Q-M346
ERS3189357	SI-47	Sidon	medieval	–	26722544	2	0.4	44	male	H2a5	R-M269
ERS3189343	SI-53	Sidon	medieval	1025–1154	266974394	19	4.4	387	male	T2	R-CTS300
ERS3189333	QED-2	Qornet ed-Deir	Roman	244–400	71266615	18	1.31	127	male	T1	T-CTS9882
ERS3189335	QED-4	Qornet ed-Deir	Roman	426–632	52457527	14	0.84	78	female	U3b	N/A
ERS3189338	QED-7	Qornet ed-Deir	Roman	237–389	95424013	22	1.55	164	female	HV1b	N/A
ERS3189339	QED-9	Qornet ed-Deir	Roman	–	154883416	58	3.14	415	female	H	N/A
ERS3189342	QED-12	Qornet ed-Deir	Roman	–	18591641	6	0.33	29	female	H2a5	N/A

a Calibrated radiocarbon date range

b Genetically determined

We combined our 13 remaining ancient samples with published ancient and modern data, creating three datasets. The HO set included 2,583 modern humans genotyped on the Human Origins array^17^, ^18^, ^19^ plus 300 ancient individuals^6^, ^18^ and consisted of 590,301 SNPs. The SGDP set included 78 modern individuals (West Eurasians and Mbuti) from the Simons Genome Diversity Project^20^ whole-genome sequenced to high coverage, plus 300 ancient individuals and consisted of 939,107 SNPs. The 1000GP set consisted of 311 modern individuals (CEU, CHB, YRI) from the 1000 Genomes Project phase 3^21^ plus 100 modern Greek (GRK) and 99 modern Lebanese,^6^ all whole-genome sequenced to ∼8× coverage and consisting of ∼80 million SNPs.

**Figure 1:**
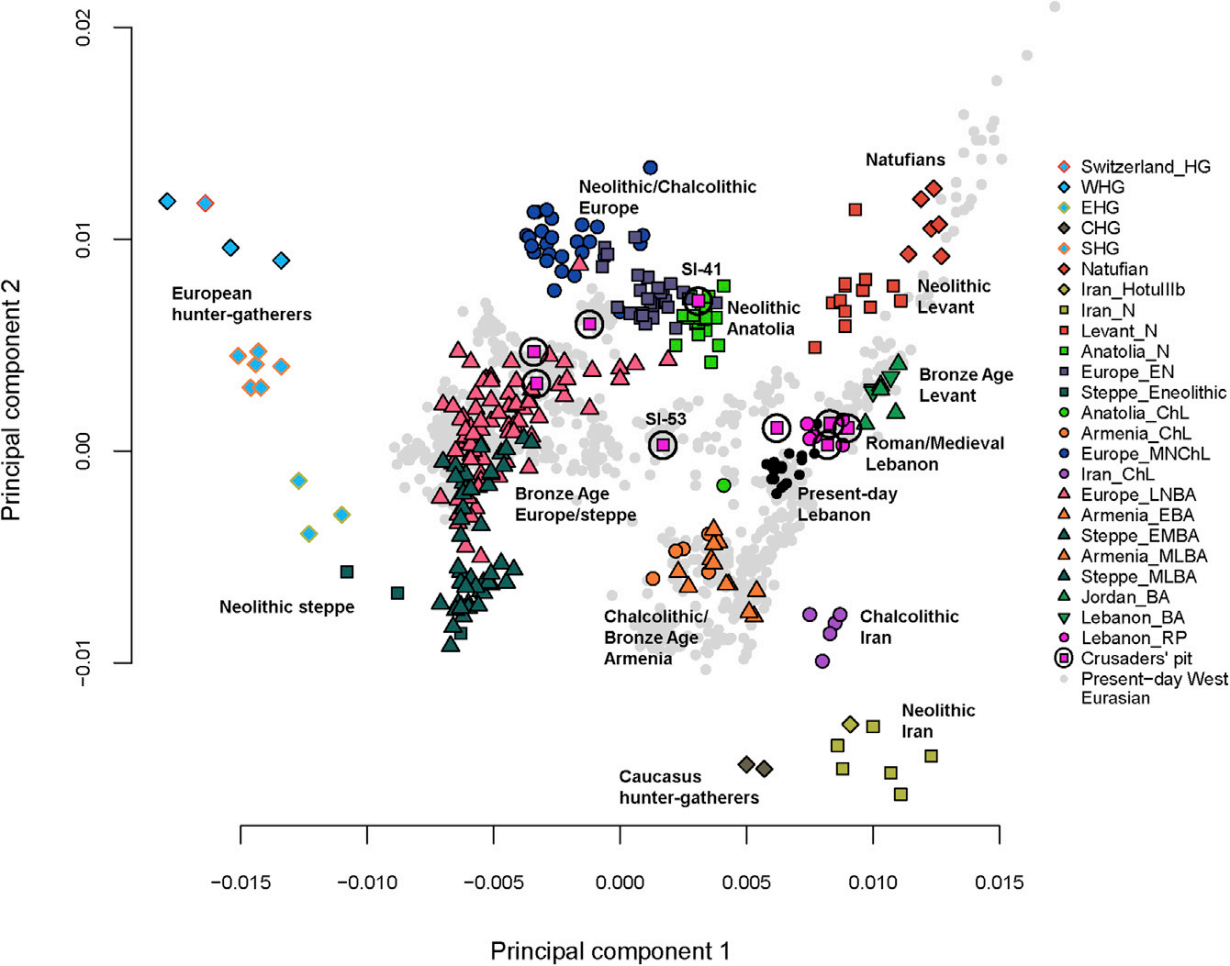
Principal Components Analysis of West Eurasians Eigenvectors were inferred using present-day populations (gray points) and the ancient samples (colored shapes) were projected onto the plot. The new ancient samples from the Roman period (Lebanon_RP) are represented by solid pink circles and the new ancient samples from the Crusaders period (Crusaders’ pit) are shown in pink squares with black circles. In addition, two samples discussed individually in the text are annotated with their IDs (SI-41 and SI-53) on the plot.

We first identified the biological sex of our samples by determining the ratio of sequences aligning to the X and Y chromosomes^22^ and found that all individuals in the Crusaders’ pit were genetically males. We then projected the ancient Lebanese samples onto a principal component analysis (PCA)^23^ plot based on modern West Eurasians of the HO dataset (Figures 1 and S5). The PCA plot revealed a previously described^6^, ^18^ structure differentiating between Europeans and Near Easterners on the first principal component with a cline in both regions over the second component. We found that all individuals from Lebanon’s Roman period (Lebanon_RP) clustered with Near Easterners and were close to present-day Lebanese. In contrast, the Crusaders’ pit individuals were more diverse and we classified them into three groups based on their PCA position. First, a group of four individuals appeared to be local Near Easterners since they clustered with the Roman period and present-day Lebanese. Second, three individuals appeared to be Europeans and clustered with different European populations (two clustered with Spaniards and were close to Basque, French, and Northern Italians, and the third clustered with Sardinians). Third, two individuals appeared to have an intermediate position between Europeans and Near Easterners: individual SI-41 overlapped with Neolithic Anatolians on the PCA and was distant from any modern West Eurasian population, and individual SI-53 overlapped with Ashkenazi Jews and South Italians.

**Figure 2:**
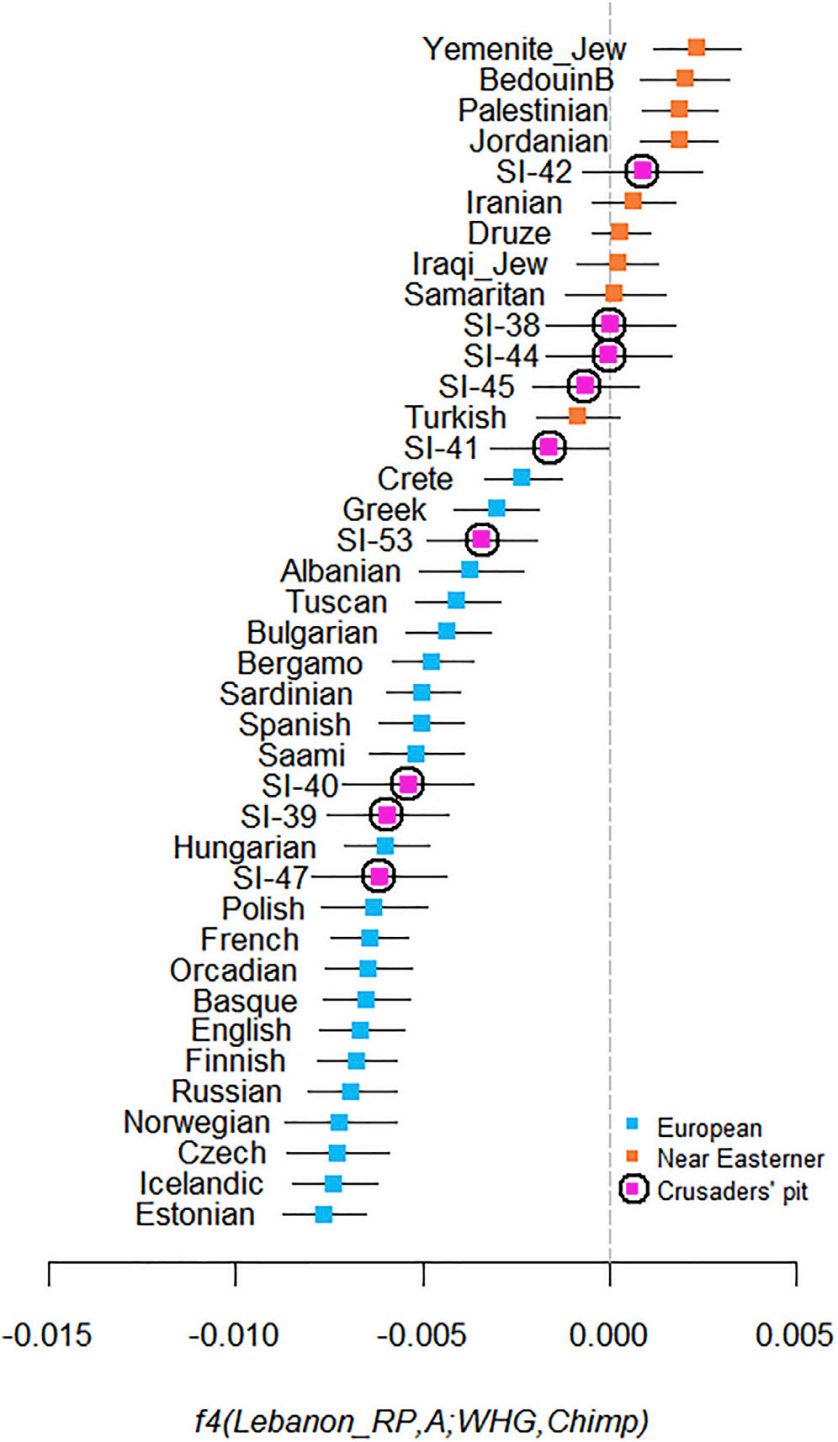
Contrast between Europeans and Near Easterners from Their Genetic Affinity to WHG The statistic *f4*(*Lebanon_RP, A; WHG, Chimpanzee*) is significantly negative when *A* is a European population. The ancient individuals from the Crusaders’ pit are represented by pink squares with black circles. We plot the *f4* statistic value and ±3 standard errors.

Therefore, the PCA results point to a diverse origin for the individuals buried in the Crusaders’ pit. We sought to confirm these results by testing the genetic affinity of the individuals to West European Hunter-Gatherers (WHG), who contributed ancestry to all Europeans but not to Near Easterners.^17^ We used the SGDP set to compute^19^ the statistic *f4(Lebanese_RP, A; WHG, Chimpanzee)*. This is expected to be negative when *A* is European because of the excess of WHG ancestry in Europeans compared with the Roman period Lebanese, but should not be significantly different from zero when *A* is a Near Easterner. As expected, we found a significant contrast between Europeans and Near Easterners from their genetic affinity to WHG (Figure 2), confirming the diversity of the Crusaders’ pit individuals: three were Europeans, four were Near Easterners, and the two who had ambiguous ancestry on the PCA appear, based on the f-statistic, to have had European ancestry but with less WHG ancestry than the other Europeans found in the pit.

Finding Europeans in the burial supports the observations from archaeology suggesting that the buried individuals could have been Crusaders. But we also found Near Eastern individuals buried in the same pit along with the Europeans. Two possible scenarios could explain this finding. (1) Following a battle between Crusaders/Europeans and a Muslim army/Near Easterners, the dead from both sides were buried in the same pit. (2) Alternatively, the Crusaders recruited local Near Easterners into their army and thus the burial was for individuals who were all fighting with the Crusaders. The historical records explain that the attacks on the Crusaders were led by Muslim armies mostly recruited from Syria, Iraq, Egypt, Turkey, and Bedouin tribes in the region. But we find the statistic *f4(Lebanese_Christian, A; Lebanon_MP, Chimpanzee)* is always positive when *A* is any Near Eastern population (Figure S6), suggesting that the present-day Lebanese Christians are genetically one of the closest populations to the Near Eastern individuals found in the pit. These results support historical records on local Christian integration into the Crusaders’ social structure including the military, with indigenous Christians joining the Crusaders as troops or becoming marshals and knights.^4^ However, we should note here that we are making an assumption that 800 years ago the medieval Near Easterners were already genetically structured in a way that allows us to differentiate between the different medieval populations in the region, i.e., a medieval Lebanese Christian could be genetically differentiated from a medieval Bedouin. Our tests (see below) suggest that genetic structure between the Lebanese and other Near Easterners could have existed more than 800 years ago but genetic structure between the Lebanese religious groups is probably more recent and therefore our tests might not be able to unambiguously differentiate between the Lebanese Christians and the Lebanese Muslims who lived during the medieval period.

**Figure 3:**
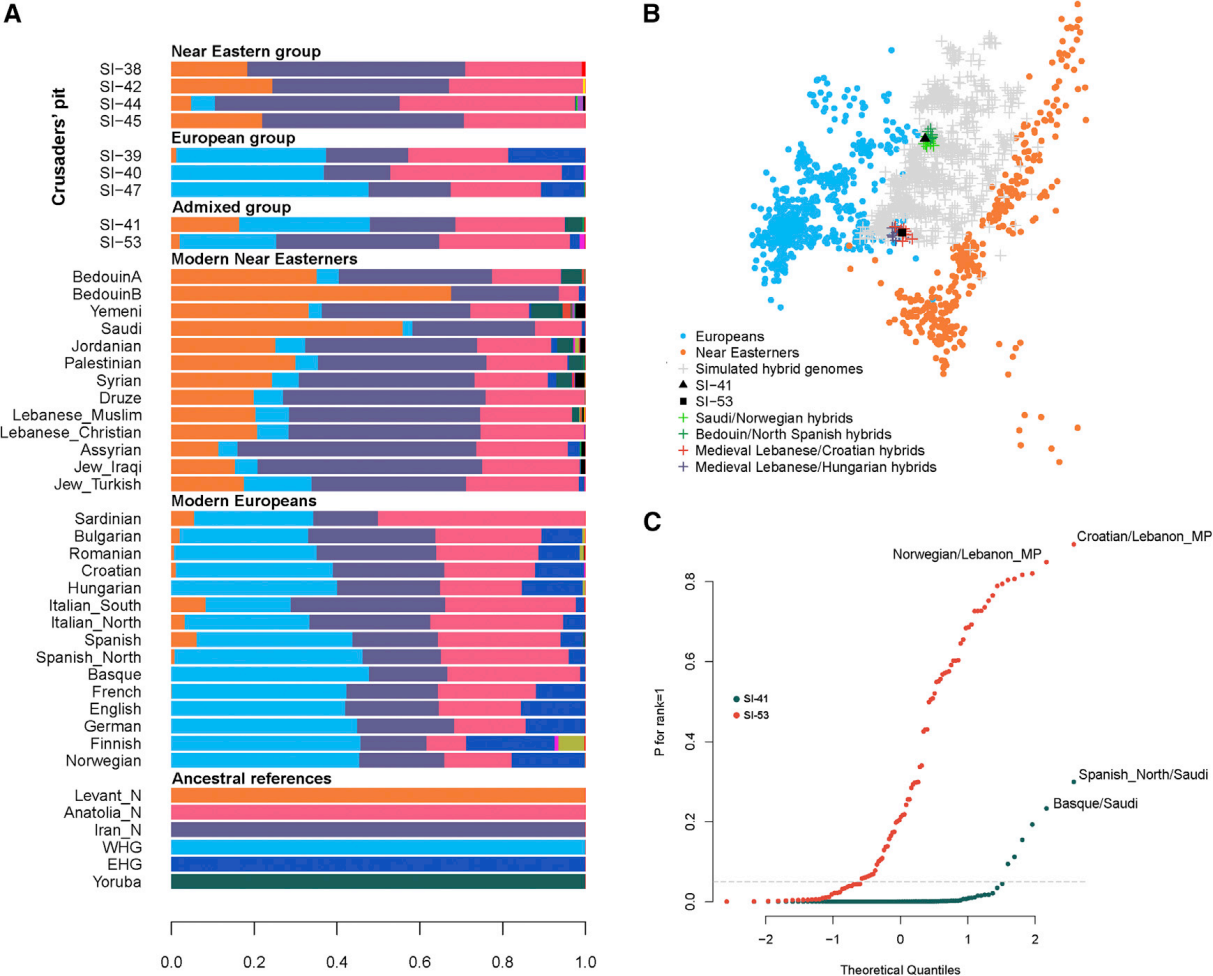
Admixed Individuals in the Crusaders’ Pit (A) ADMIXTURE plot of the HO set using ∼80,000 transversions and showing results from K = 17 in a supervised run with references: Anatolia_N, Iran_N, EHG, WHG, Levant_N, Atayal, Chipewyan, Eskimo, Hadza, Mala, Mbuti, Nganasan, Onge, Papuan, Quechua, Taa_West, and Yoruba. We show results from the ancient individuals from the Crusaders’ pit in addition to the median of the ancestry proportions found in modern populations. (B) The position on the PCA of two individuals found in the Crusaders’ pit can be explained by admixture. Simulated (first generation) hybrid samples (gray crosses, with some highlighted in color as indicated on the figure) consisting of genomes with positions represented by an allele from a Near Easterner (orange circles) and an allele from a modern European (blue circles) show intermediate positions on the PCA and overlap with the ancient individuals SI-41 and SI-53. (C) Individuals SI-41 and SI-53 can be successfully modeled as mixture of Europeans and Near Easterners using *qpAdm*. We show the p values for rank = 1 and annotate the models with the top values.

In addition to the three Europeans and four Near Easterners found in the burial, there were two individuals (SI-41 and SI-53) who we could not associate with any specific group. We consequently wanted to explore the possibility that these individuals’ particular pattern on the PCA might be due to their genomes being admixed. Therefore, we first ran an ADMXITURE^24^ analysis (Figure 3A) which confirmed the presence of three genetically different groups in the Crusaders' pit with SI-41 and SI-53 having intermediate ancestry compositions compared with the other ancient individuals. Next, we simulated hybrid diploid genomes by selecting pairs of individuals, one from the Near East and the second from Europe and we picked a random allele from each individual. We then projected the simulated hybrid genomes onto the PCA plot, looking for hybrid combinations that overlapped with individuals SI-41 and SI-53. We found that a mixture between a medieval Lebanese and a Croatian or a medieval Lebanese and a Hungarian could reproduce the PCA position of SI-53, while a mixture between a Saudi and a Norwegian, or a Bedouin and a Northern Spanish, could reproduce the position of SI-41 (Figure 3B). We assessed these results with formal tests using *qpAdm*^25^ and setting the source populations for SI-41 and SI-53 as pairs of populations selected from two groups from the HO set: (1) Medieval Lebanese, Lebanese Christian, Syrian, Palestinian, Assyrian, Iraqi Jew, Turkish Jew, Ashkenazi Jew, Saudi, and Bedouin and (2) French, Italian, Spanish, Basque, English, Norwegian, German, Croatian, Hungarian, Romanian, and Ashkenazi Jew. Then we selected a set of outgroups that are related differently to the source populations: Ust’-Ishim, Kostenki 14, MA1, Onge, Papuans, Chukchi, Karitiana, Eastern hunter-gatherers (EHG), WHG, Natufians, Caucasus hunter-gatherers, Neolithic Iranians, Neolithic Anatolians, and Neolithic Levantines. We found that both SI-41 and SI-53 can be modeled as mixtures of European and Near Eastern ancestries. For individual SI-41, the best-supported model is a descent from a Near Easterner related to a Bedouin or a Saudi and a European related to Northern Spanish or Basques (Figure 3C, Table S3). For individual SI-53, the best-supported models involved a Near Easterner related to medieval Lebanese, Lebanese Christians, or Jews and a European who can be from diverse origins (Figure 3C, Table S4). We investigated the robustness of our *qpAdm* inferences by testing, similarly to SI-41 and SI-53, the European individuals from the Crusaders’ pit and, as expected, we found that most of their ancestry was derived from Europeans in contrast to SI-41 and SI-53 (Table S5). Individual SI-53 clustered on the PCA with Ashkenazi Jews, Sicilians, and Southern Italians and therefore we wanted to test whether SI-53 could have descended directly from one of these populations who were previously reported to be admixed,^26^, ^27^ with the implication that admixture in SI-53 could then have occurred before the Crusaders’ time and in Europe instead of in the Near East. However, our results (Table S6) show that the Europeans have in general a distinct admixture pattern from the one observed in SI-53; among the populations tested, Ashkenazi Jews’ Near Eastern ancestry is mostly related to Near Eastern Jews, Sicilians’ European ancestry is related to Italians, and Southern Italians have Northern Italians as top sources of their European ancestry. We note here that the reference populations tested are not necessarily the precise parental populations; any genetically equivalent populations could have been involved, but the admixture patterns in SI-41 and SI-53 suggest our data (1) provide direct genetic evidence of admixture between Crusaders and Near Easterners, (2) consequently show admixture could have been common (at least in Sidon), as two out of nine sequenced individuals were admixed, (3) show that admixture with the Near Easterners was not limited to only Levantine Christians but also involved people genetically related to Saudis and Bedouins, and (4) provide support for our previous assumption that 800 years ago the Near Easterners were already genetically structured in a way that allows us to differentiate coastal Levantines from inland people such as Bedouins and Saudis.

Next, we analyzed the variations on the Y chromosomes^28^ of all ancient males and the mtDNA genomes^29^ of all ancient individuals and determined the respective haplogroups, which can provide information on the place of origin of the paternal and maternal lineages. All ancient individuals assigned in this work as either European or Near Eastern had Y and mtDNA haplogroups that reflected this ancestry, i.e., Europeans had Y-haplogroup R1b and mtDNA-haplogroups H or U5, while the Near Easterners had Y-haplogroups E, T, J, and Q and mtDNA-haplogroups J1 or HV (Table 1). However, the admixed individuals SI-41 and SI-53 had Y-haplogroup R1b-P312 typically found in European males and mtDNA haplogroups HV0 and T2 present in both Europe and the Near East. In particular, SI-41 carried the DF27 lineage, which is highly prevalent in Iberia (up to 70% of males in Basque) and rare elsewhere,^30^ supporting our previous results from the autosomal variants that this individual descended from a European related to Northern Spanish or Basques. The combined results from the Y and mtDNA haplogroups suggest that SI-41 and SI-53 possibly had a European father and a Near Easterner mother, but a more complex admixture scenario, where the parents themselves are admixed, could also produce this ancestry pattern. Our tests^31^ on the X chromosome were inconclusive about the ancestry of SI-41 and SI-53, probably because the reduced sequencing coverage and diversity compared with the autosomal genome make them less powerful (Figure S7).

Our data suggest that admixture occurred in Lebanon during the Crusaders’ time; therefore, we sought to investigate the consequences of this admixture for the genomes of the Lebanese by tracking the genetic changes that occurred in Lebanon before and after the Crusades. We started by using the SGDP set to compute the statistic *f4(Lebanon_BA, Lebanon_RP; Ancient West Eurasian, Chimpanzee)* which quantifies the genetic changes related to West Eurasians that occurred in Lebanon between the Bronze Age and the Roman period. We found there was an increase in Eurasian hunter-gatherer and Steppe population ancestry in Lebanon after the Bronze Age (Figure S8A), which provides direct evidence for our previous inference that this Eurasian ancestry in the Levant predates both the Crusaders and the Roman period.^6^ Next, we computed *f4(Lebanon_RP, Lebanon_MP; Ancient West Eurasian, Chimpanzee)* and found that the statistic is not significantly different from zero for any Ancient West Eurasian tested, thus indicating that there were no significant genetic changes between the Roman period and the medieval period in Lebanon (Figure S8B). We then tested *f4(Lebanon_MP, Lebanese_Christians; Ancient West Eurasian, Chimpanzee)* and *f4(Lebanon_MP, Lebanese_Muslims; Ancient West Eurasian, Chimpanzee)*; there were no significant genetic differences between medieval Lebanese and present-day Lebanese Christians (Figure S8C), but we found that Lebanese Muslims had significantly lower genetic affinity to West Eurasians (Figure S8D). This genetic change in the Lebanese Muslims could potentially be a result of gene flow from a population genetically distant from West Eurasians. We investigated this possibility by computing *f3*(*Lebanese_MP, A; Lebanese_Christians*) and *f3*(*Lebanese_MP, A; Lebanese_Muslims*), which test whether the Lebanese groups descended from a mixture between medieval Lebanese and another population. We found that Lebanese Christians cannot be modeled in this way (Figure S9A), but Lebanese Muslims had negative *f3*-statistics when *A* was an African or a Central/East Asian population (Figure S9B), indicating that they are admixed from these sources. We confirmed these results by analyzing the 1000GP set using *rarecoal-tools*^32^ which identifies genetic ancestry using rare variants and thus complements the low sensitivity of the f-statistics to detect admixture when the ancestral mixing fractions are small. We find an enrichment of African and East Asian rare alleles in the Lebanese Muslims compared with the Lebanese Christians, but we found no substantial differences related to their European ancestry (Figure S10). Therefore, our results suggest that the genome-wide genetic legacy of the Crusaders cannot be observed today in the Lebanese even though the Lebanese ancestors have admixed with the Crusaders—as it is evident from the genome of individual SI-53. We propose that admixture during the Crusaders’ time was not widespread enough to leave genetic traces quantifiable in the present-day populations. A shared recent genomic history between Europeans and Near Easterners also contributes to rapidly diluting a specific genetic signal of gene flow from the Crusaders. For example, the Neolithic Anatolians and Bronze Age steppe people, who contributed ancestry to the Europeans,^17^ were themselves a mixture of populations that included Neolithic Levantines and Chalcolithic Iranians^18^ who were also major contributors to the genetics of Near Easterners.^6^ We show by simulating hybrid genomes of decreasing European ancestry that a Lebanese who had a French great grandparent cannot be distinguished—by their genetic relationship to ancient West Eurasians—from a Lebanese who did not have admixed ancestors (Figure S11). Thus our data highlight the power of aDNA in informing on past events that would be otherwise undetectable from modern autosomal DNA alone.

Remarkably, the significant genetic changes in the Lebanese following the Crusades appear to have been not from the Crusaders and were not in the Lebanese Christians, but were mostly restricted to the Lebanese Muslims. We analyzed admixture LD^33^, ^34^ in the Lebanese in order to date the time when admixture occurred in their history. We found two signals; the first admixture can be detected with overlapping dates in both Lebanese Christians (850–150 BCE, Z = 6.95) and Lebanese Muslims (900 BCE–500 CE, Z = 3.02) and is consistent with finding this admixture in our ancient Roman period samples 240–400 calCE. However, the second admixture was specific to Lebanese Muslims (Figures S12A and S12B) occurring around 1550–1700 CE (Z = 3.69) and can be detected when Africans and East Asians are used as reference populations. The time of this admixture coincides with the Ottoman Turkish rule over Lebanon and we propose that the Turks, who themselves carry East Asian ancestry (Figure S12C) from their Seljuk ancestors,^35^ brought this ancestry to the Lebanese Muslims. The African ancestry was introduced into the Lebanese Muslims most likely via the slave trade in the Ottoman Empire and the prohibition of non-Muslims from owning slaves during this period.^36^

In this report, we have presented new whole-genome sequence data from ancient individuals who lived during times when major historical events were unfolding in the Near East. Our samples from the Crusaders period are evidence of a remarkable genetic diversity that coexisted in this region—Europeans, Near Easterners, and their mixed descendants—but this heterogeneity was transient in the genomic history of the Near East, since, with the exception of some Y chromosome lineages, present-day populations derive most of their ancestry from local people who preceded the Crusades. Ancient DNA from the warm Near East is still problematic to retrieve, and in addition our samples from the Crusaders’ pit were crudely buried and partly burned, but our study shows that recovering such aDNA is feasible and that the historical and genetic insights from it are exceptional and not possible from modern DNA alone.

## Declaration of Interests

The authors declare no competing interests.

## Accession Numbers

Raw sequencing reads for the ancient individuals are available through the European Nucleotide Archive (ENA) under accession number ERP114192. Aligned sequences and genotypes can be obtained from the corresponding authors.

## Web Resources

European Nucleotide Archive (ENA), https://www.ebi.ac.uk/ena

## Supplemental Data

Document S1. Figures S1–S12 and Tables S1–S6

Document S2. Article plus Supplemental Data
