# Supplementary material for: A Transient Pulse of Genetic Admixture from the Crusaders in the Near East Identified from Ancient Genome Sequences
Source: Am J Hum Genet. 2019 Apr 18;104(5):977–84. doi: 10.1016/j.ajhg.2019.03.015 (PMC6506814; doi:10.1016/j.ajhg.2019.03.015)
Supplement: Document S1. Figures S1–S12 and Tables S1–S6 [file mmc1.pdf]

The American Journal of Human Genetics, Volume *104*

Supplemental Data

A Transient Pulse of Genetic Admixture from the Crusaders in the Near East Identified from Ancient Genome Sequences

Marc Haber, Claude Doumet-Serhal, Christiana L. Scheib, Yali Xue, Richard Mikulski, Rui Martiniano, Bettina Fischer-Genz, Holger Schutkowski, Toomas Kivisild, and Chris Tyler-Smith

Supplementary materials

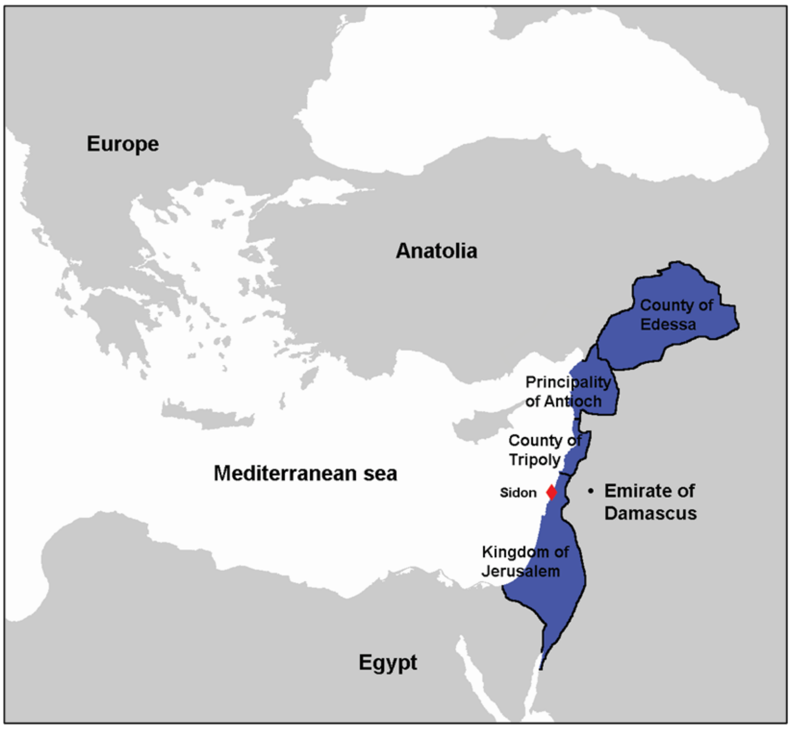

Figure S1. The Crusaders States in the Near East. Map shows the four Crusaders states in the Near East in the first half of the 12th century CE with their geographic extent colored in blue. The Crusaders samples analyzed in this study were buried in the city of Sidon (red lozenge) which was part of the Kingdom of Jerusalem.

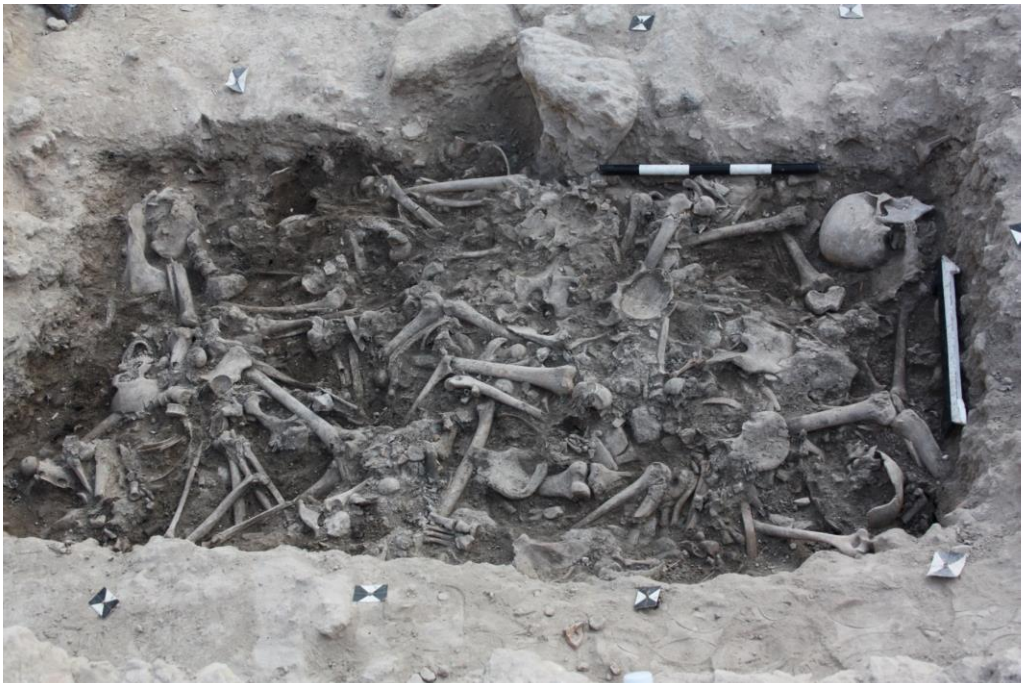

Figure S2. Burial 110 "the Crusaders' pit" general view. Between 2009 and 2010, archaeological deposits representing two mass burials were excavated in Square 47 at College Site, Sidon, Lebanon, within what is now identified as the fortification dry moat which surrounded the eastern side of the medieval city of Sidon. The larger of the two deposits, known as burial 110, consisted of a rectilinear pit measuring a maximum of 2m east-west and 1.3m north-south. The pit had been cut into the Late Iron Age deposits marking the base of the fortification ditch, to a reasonably consistent depth of 30cm. The fill of the pit consisted mainly of a mixed assemblage of incomplete, partially articulated and disarticulated, commingled human skeletal remains with no clear organization of the elements or body parts evident. Alongside the human remains, a variety of artefacts was recovered including a number of Frankish buckles and a single base silver denaro coin dating to 1245-1250 CE, together with other artefacts consistent with the period of the Crusades. Seven individuals were successfully whole-genome sequenced from this pit: SI-38, SI-39, SI-40, SI-41, SI-42, SI-45, and SI-47. The second, smaller deposit of human skeletal remains, known as burial 101, lay just outside pit of burial 110, approximately 50cm to the northeast. This group also consisted of partially articulated remains but exhibited a greater degree of completeness of with more whole limbs present and an almost complete upper body. No cut for a pit was identified associated with burial 101 and it's possible it may have originally been larger or more extensive, with subsequent truncation occurring in the intervening period between the Crusades and the modern archaeological excavation. An alternative interpretation is that this smaller group of remains represents a collection of larger body parts placed close to burial 110 in anticipation of their inclusion within the pit, but which process did not ultimately take place for unknown reasons. One individual (SI-44) was successfully whole-genome sequenced from this pit. Together the burials 101 and 110 consisted of human remains representing a minimum of twenty-five individuals, exhibiting evidence of multiple perimortem traumata to multiple individuals, indicating the remains belonged to a group who likely died in a single violent event. An additional sample within this study (SI-53) was taken from an isolated skull, known as context (10153). This skull, which was articulated with several upper cervical vertebrae but not directly associated with any other remains, was also recovered from within the fortification ditch, approximately 5m to the southwest of burial 110, close to the base of a round tower, flanking what is interpreted as a gated entrance way across the ditch, just to the south. The radiocarbon age of SI-53 is within the timeframe of the Crusaders' presence in the region but appears to predate the time of the rest of the samples from burial 110. Additional archaeological research in the future will aim to understand if SI-53 was directly associated with the event of burial 110 or was from a distinct event during the Crusader period.

A B

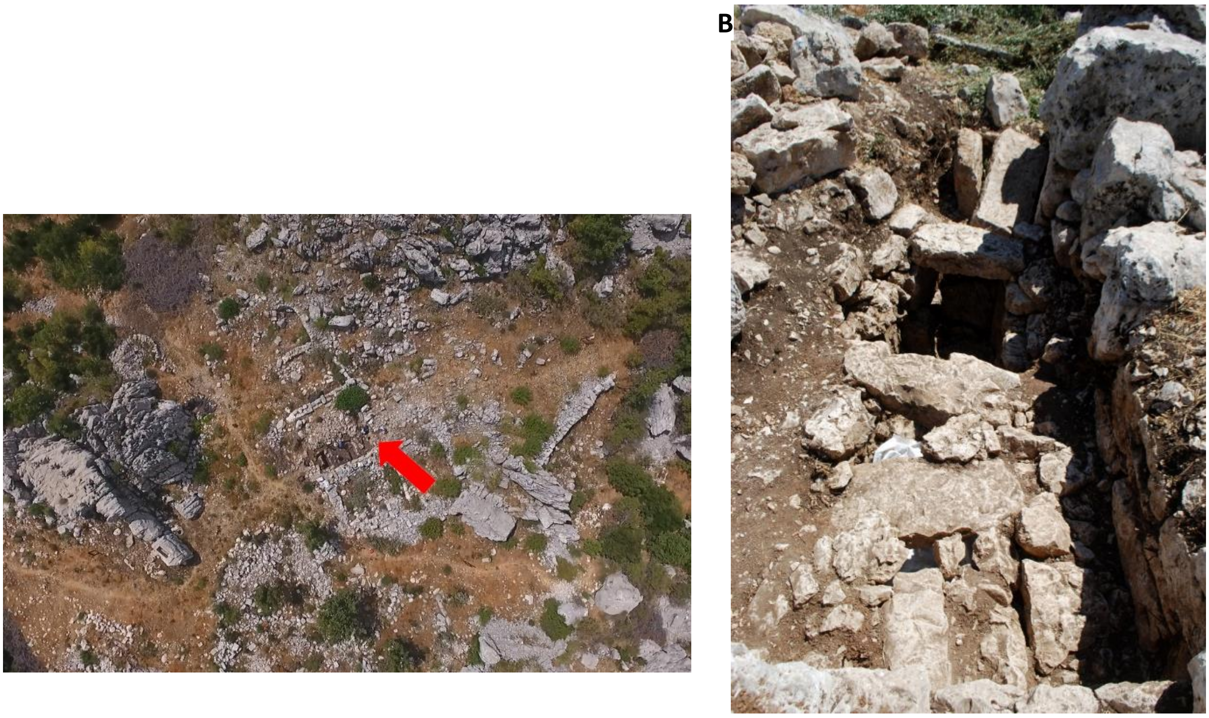

Figure S3. The archaeological site of Qornet ed-Deir. The site of Qornet ed-Deir, located in the Jabal Moussa Biosphere Reserve in Mount Lebanon, is a naturally fortified rocky outcrop overseeing one of the communication roads leading from the coast to Afqa and over the Lebanon Mountains into the Beqaa. The site was excavated in 2017 and it is adjacent to remains of a building with well-built massive walls which was probably a way station or military post during the Roman period. At the top of the site, several multi-room buildings are preserved, and the settlement history uncovered so far spans from the Middle Bronze Age period to the Late Crusader period. In one of these rooms a well-built chamber tomb from the Roman period was discovered (A). The access to the tomb was from the south and closed with a vertical stone slab. Three large roof stones were preserved *in situ*, and one more fractured slabs covered the back of the chamber. After the removal of one of the middle-roof stones, it was possible to access the tomb (B). Several *craniums* were collected from this fill, and these provided the Petrous bone samples used in this study.

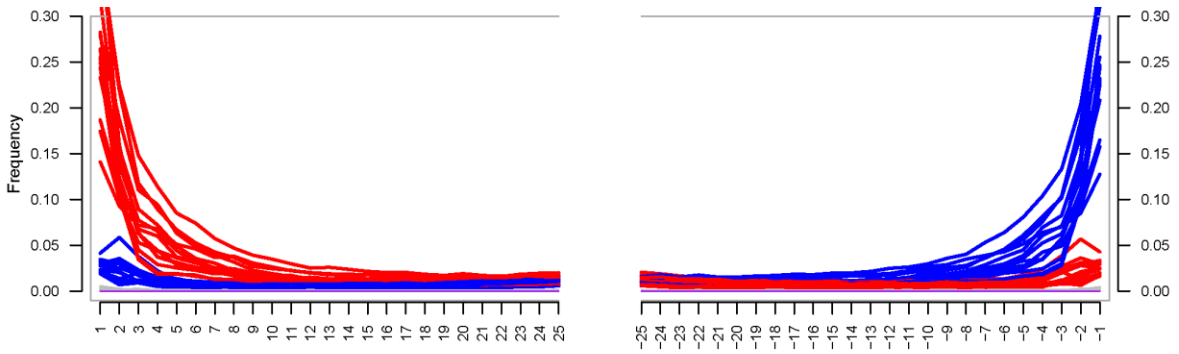

Figure S4. Post-mortem damage patterns. Base substitutions C>T from the 5' (left) and G>A from the 3' end (right) show patterns typical of ancient DNA damage for all samples sequenced in this study. Red: C to T substitutions, blue: G to A substitutions.

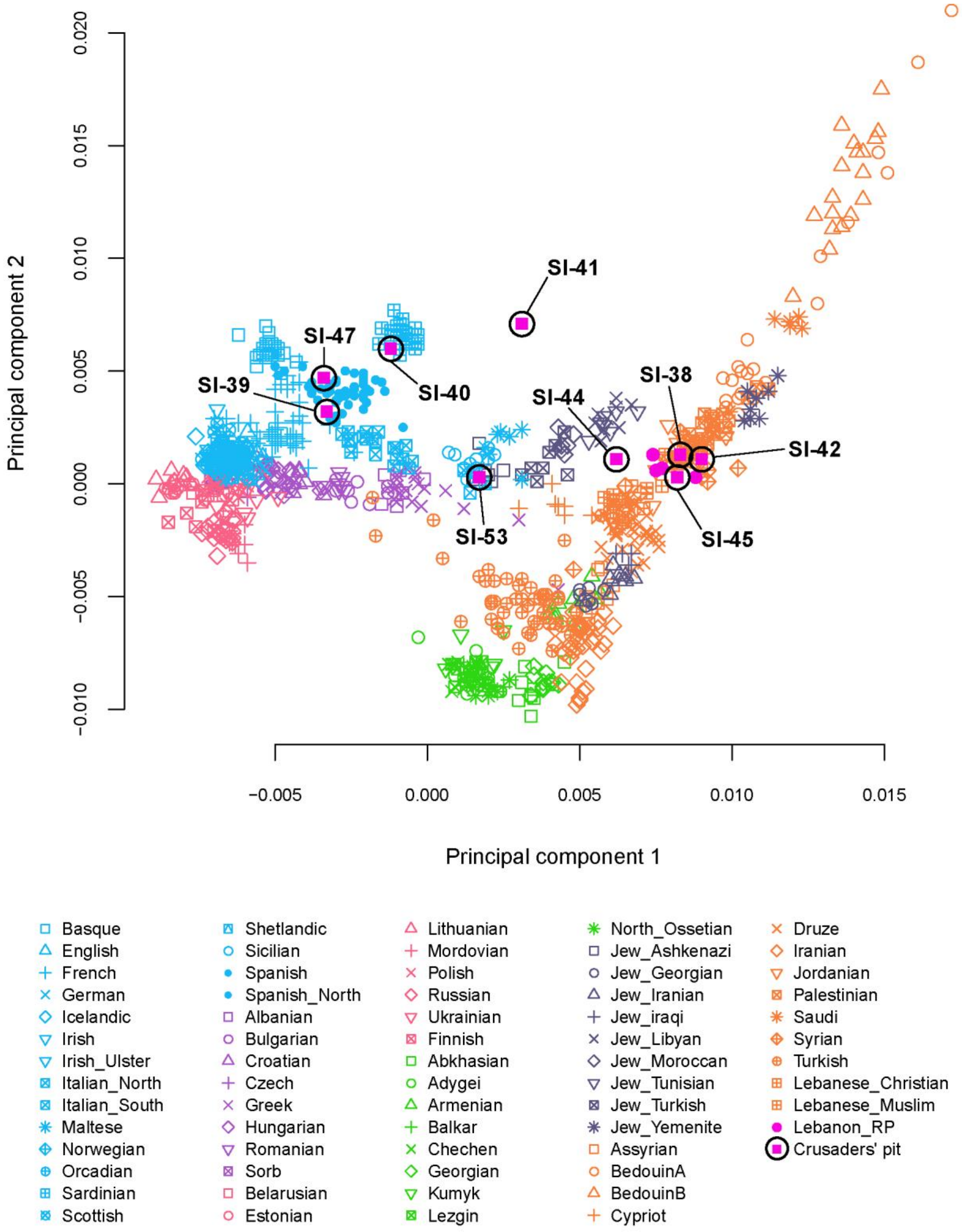

Figure S5. Principal Components Analysis of West Eurasians. Plot shows the new ancient samples in the genetic context of modern West Eurasians.

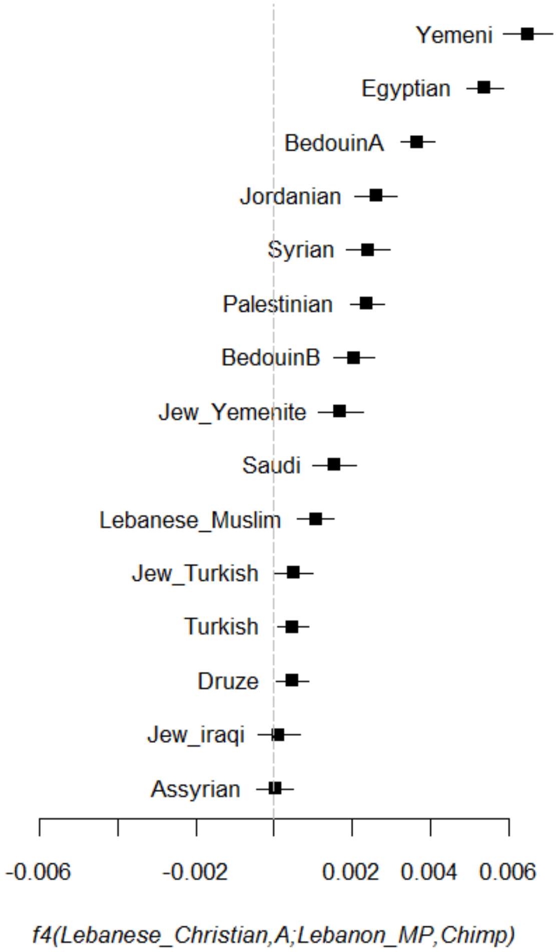

Figure S6. Testing Lebanon_MP (Near Easterners found in the Crusaders' pit) affinity to present-day Near Easterners. The statistic *f4*(Lebanese_Christian, *A*; Lebanon_MP, Chimpanzee) is always positive when *A* is a present-day Near Easterner, indicating that Lebanon_MP share significantly more alleles with the Lebanese Christians than with most other present-day Near Easterners.

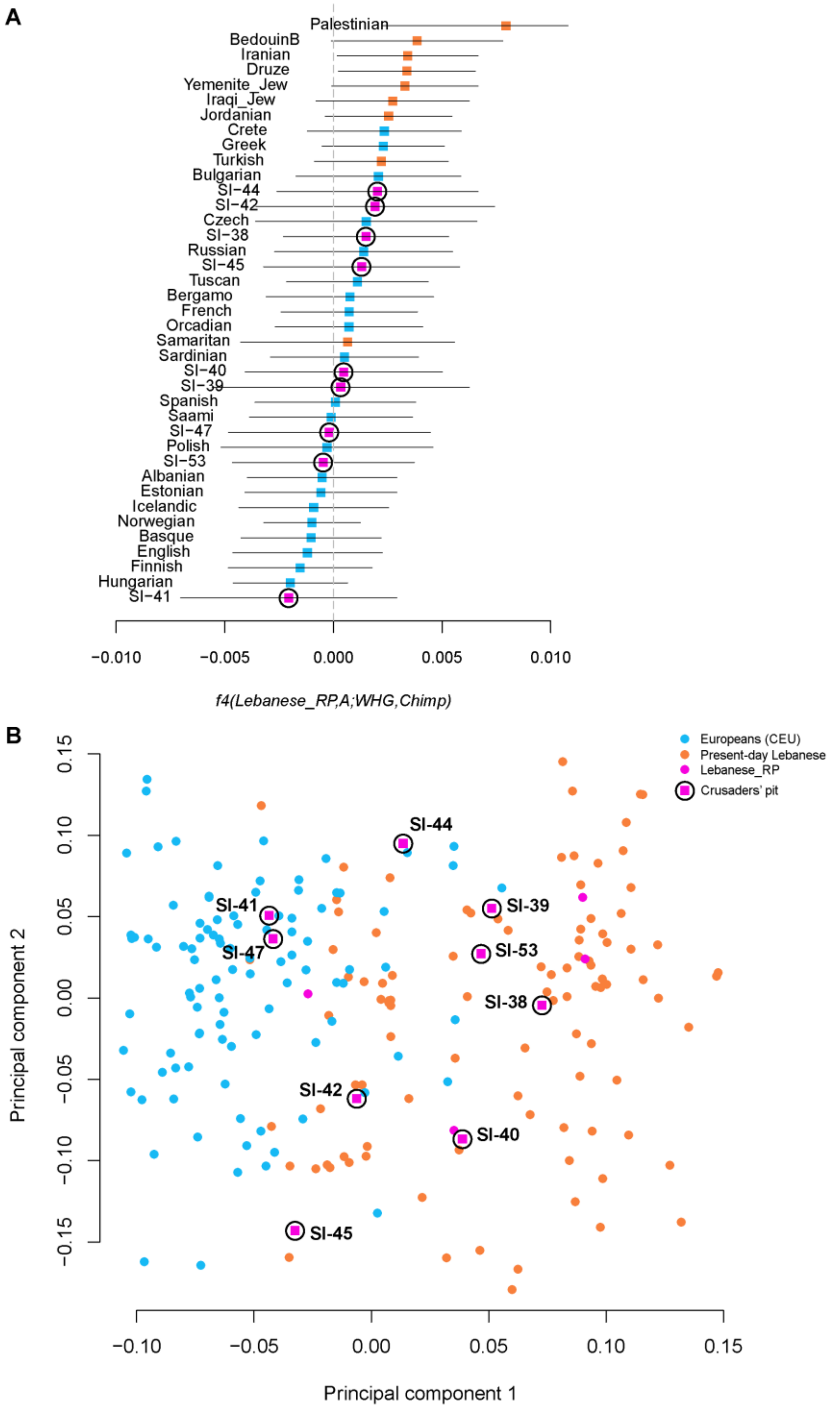

Figure S7. Diversity on the X chromosome. We explored the X chromosomal diversity in our dataset to investigate the maternal ancestry of the admixed individuals SI-41 and SI-53. We observed a reduced diversity on the X chromosome between Europeans and Near Easterners compared with the autosomal genome probably from a lower number of SNPs, a smaller effective population size and reduced depth of sequencing on the male X chromosome compared with the autosomes. (A) the statistic *f4(Lebanese_RP, A; WHG, Chimpanzee)* using ~150,000 transversions on the X chromosome in the SGDP dataset and (B) PCA run with PCAngsd using ~60,000 transversions (-minMaf 0.1) in the *1000GP* set plus the ancient individuals, both show inconclusive results on the ancestry of the ancient individuals.

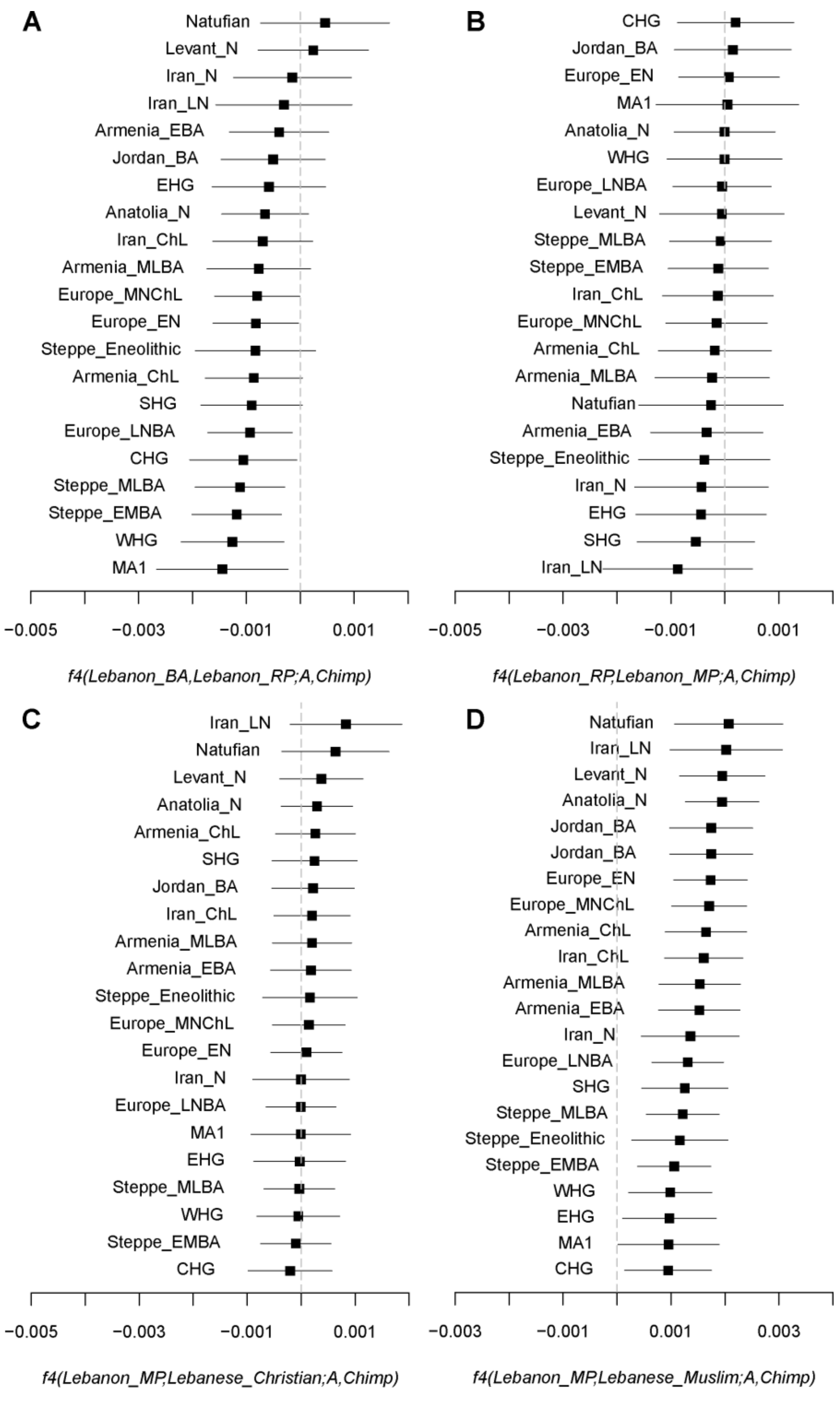

Figure S8. Genetic changes in Lebanon over time. We use *f4* statistic to detect the genetic changes in Lebanon from sources related to ancient Eurasians occurring (A) between the Bronze Age and the Roman period, (B) between the Roman period and the medieval period and (C)(D) between the medieval period and the present-day in the different Lebanese groups. We plot the *f4* statistic value and ±3 standard errors.

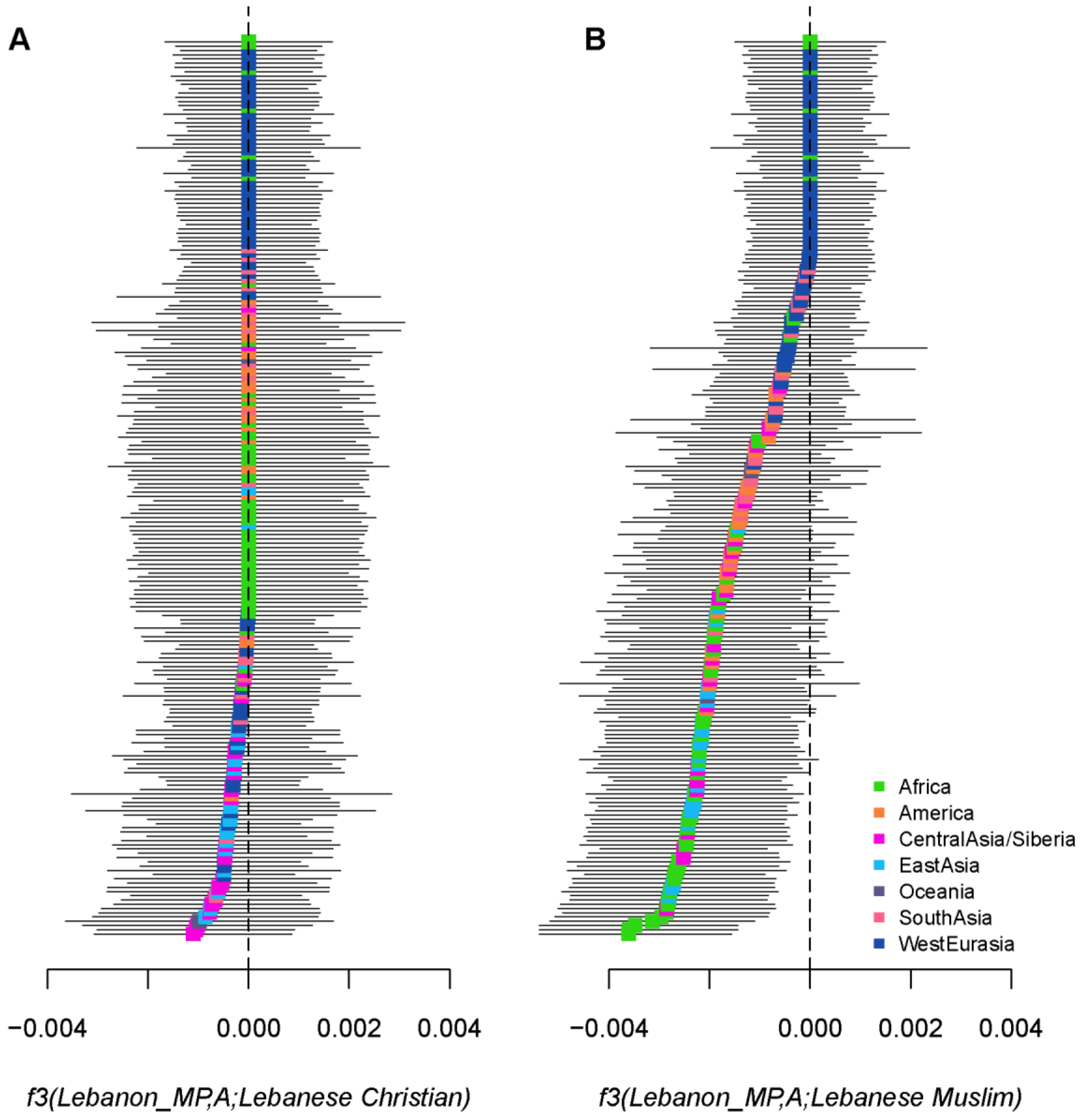

Figure S9. Admixture in the present-day Lebanese groups. We tested the Lebanese as descending from a mixture of medieval Lebanese and another population *A*. (A) The *f3*-statistic is not significantly different from zero for any population *A* when the Lebanese Christian population is set as the target of admixture; (B) but is significantly negative when Lebanese Muslim is set as the target population and *A* is an African or Central/East Asian. We plot the *f3* statistic value and ±3 standard errors.

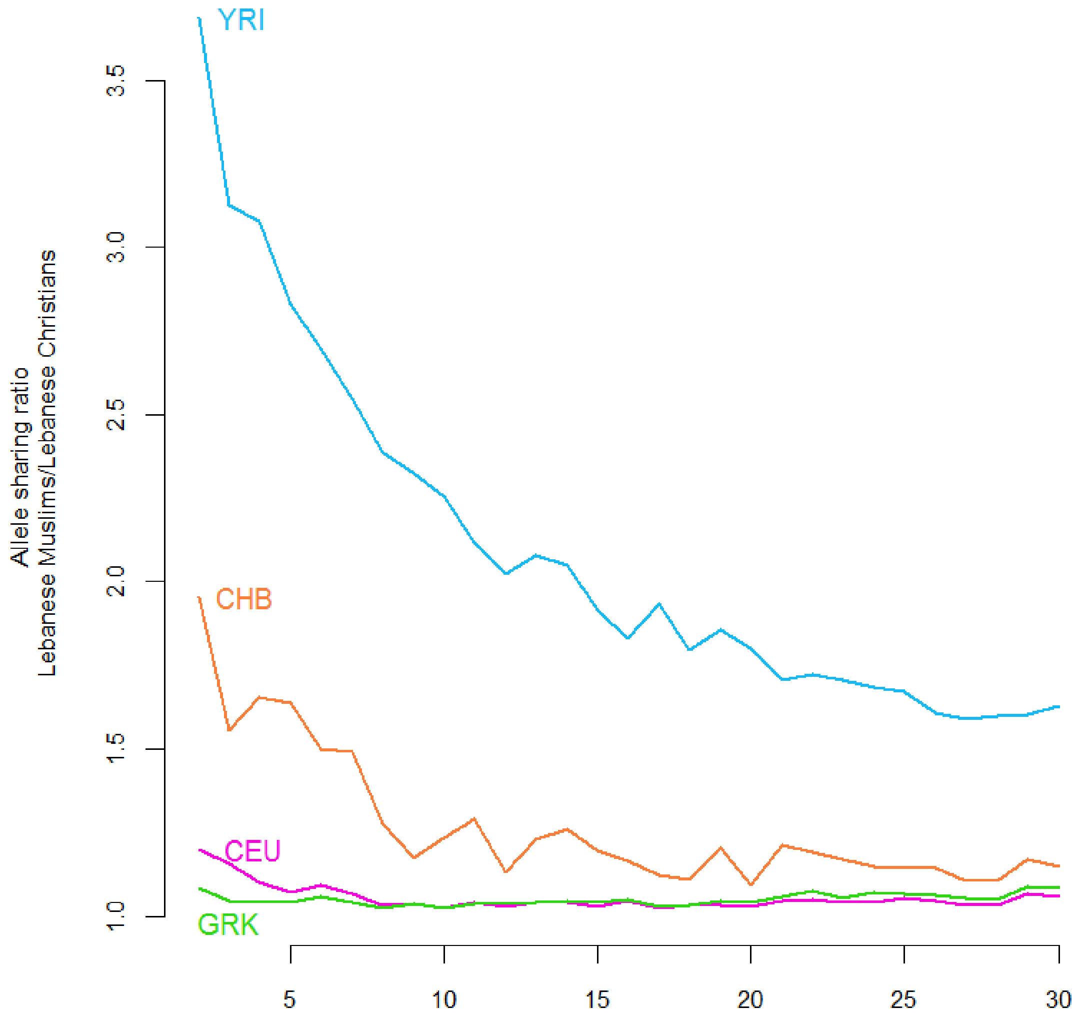

Figure S10. Rare allele sharing with the Lebanese groups. We used *bcftools* to filter for biallelic SNPs with at least one observed derived allele and maximum allele frequency of 0.05 in our *1000GP* dataset and then generated frequency sums using *vcf2FreqSum* (Schiffels et al., 2016). We set the number of individuals in each group as follows: 90 YRI (Yoruba in Ibadan, Nigeria), 90 CEU (Utah Residents with Northern and Western European Ancestry), 90 CHB (Han Chinese in Beijing, China), 90 GRK (Greeks), 32 Lebanese Christians, and 32 Lebanese Muslims. We then grouped frequency sums for all populations using *groupFreqSum* and restricted the variants to regions with high mappability with *filterFreqSum*. We generated a histogram file for each chromosome with *freqSum2Histogram* with maximum allele count of 30 per population, and combined histograms for each chromosome with *combineHistograms*. We then estimated the number of shared alleles between the Lebanese groups and other populations in the dataset at allele counts 2 to 30 and then plotted the ratio. Our data show gene flow from YRI and CHB to the Lebanese Muslims.

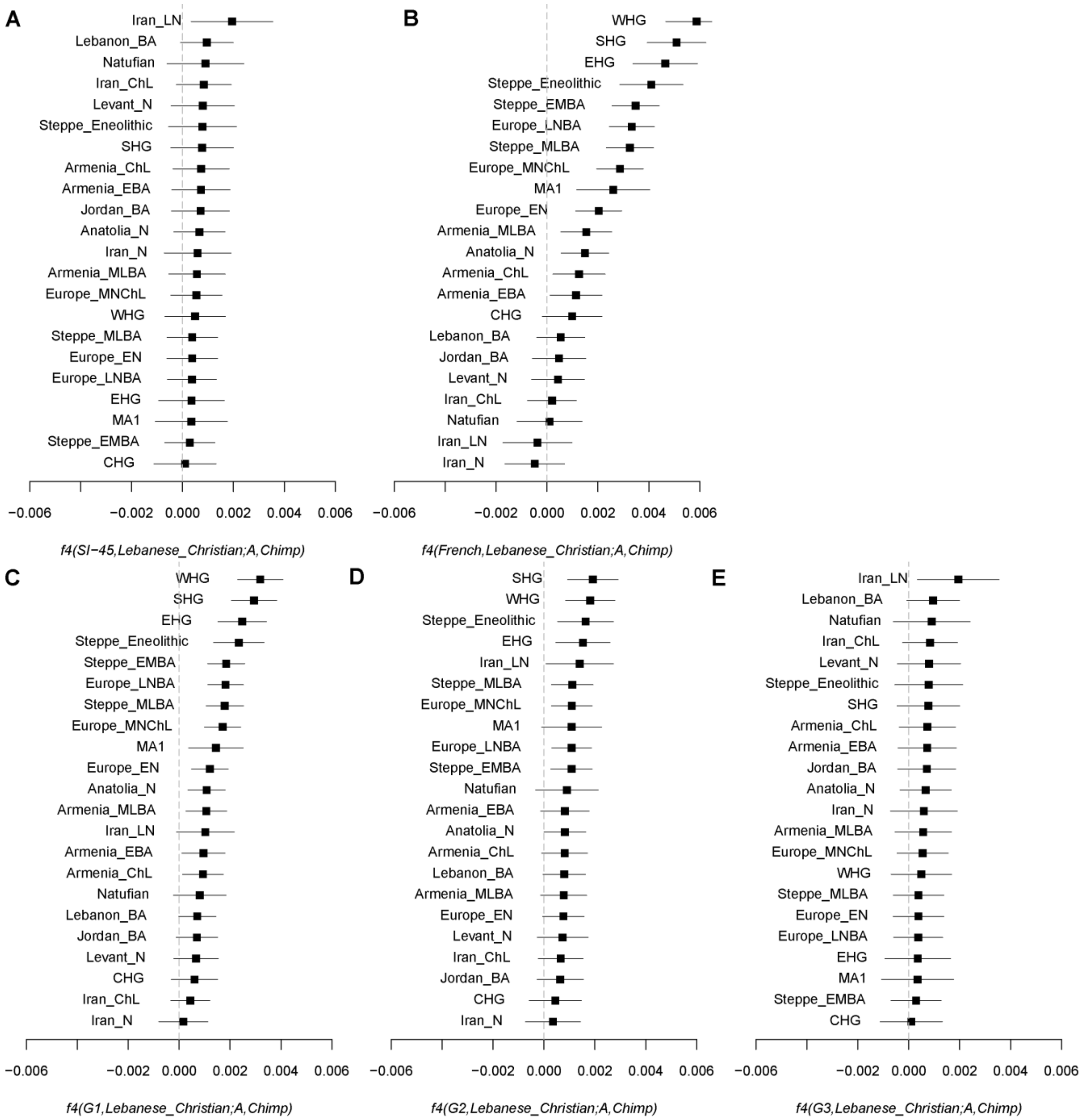

Figure S11. Differentiating admixed individuals with *f4*-statistic. (A) A medieval Lebanese (SI-45) and a present-day Lebanese Christian have almost identical genetic relationship to ancient Western Eurasians but (B) A French has more genetic affinity than a Lebanese Christian to several ancient Western Eurasian populations such as the Eurasian hunter-gatherers and the Steppe populations. (C) A simulated individual who half of his genome is from SI-45 and the other half is from a French can also be differentiated from a Lebanese based on their relationship to the ancient populations. (D) this also applies to a genome from a second generation mixing with 25% French but (E) a third generation mixed individual with 12.5% French ancestry and a Lebanese individual appear from the *f4*-statistic to have similar relationship to the ancient Western Eurasians. This insensitivity to smaller admixture proportions is due to modern Europeans and Near Easterners being descendants from genetically related ancient populations.

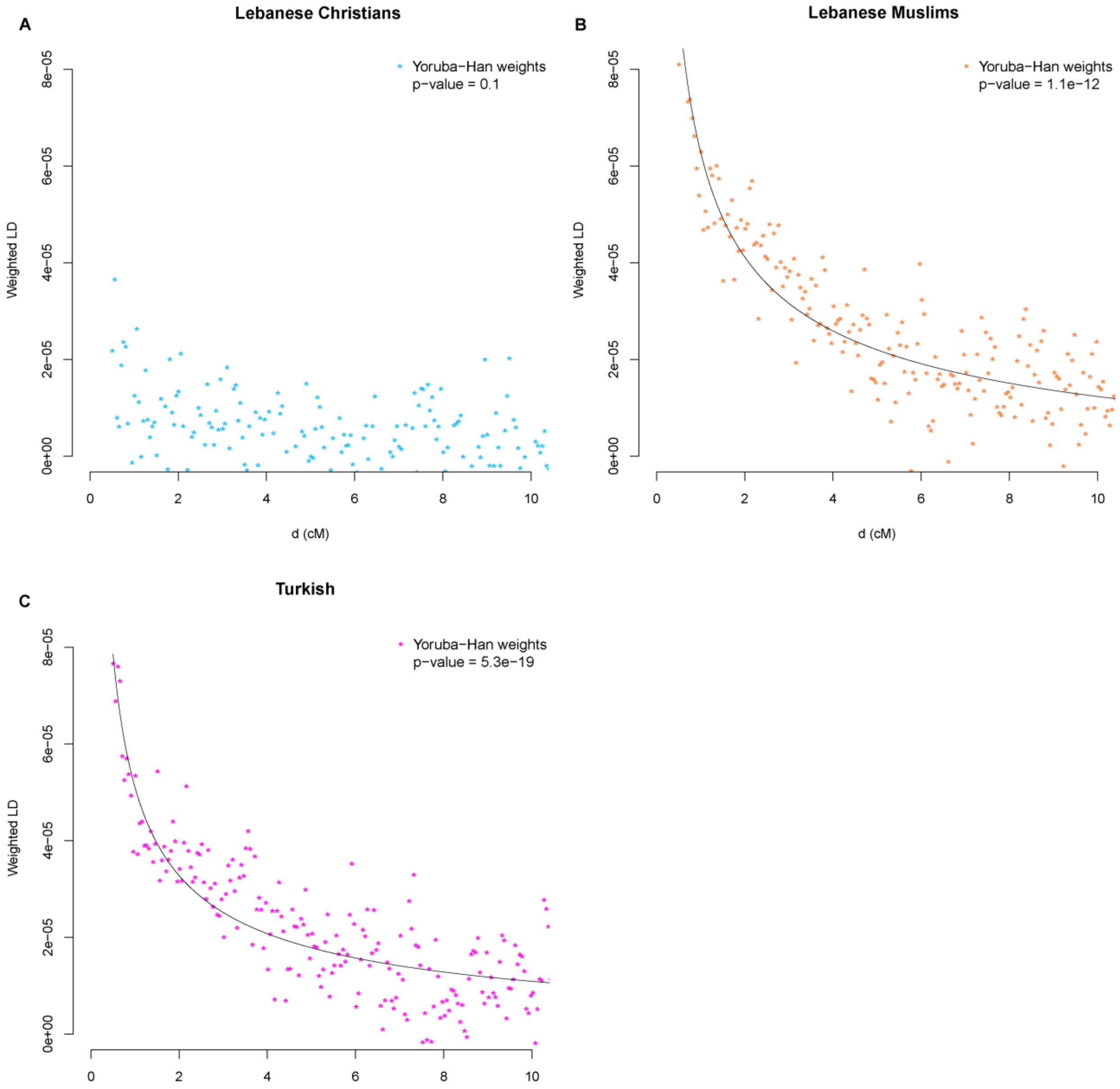

Figure S12. Weighted LD curves using Yoruba and Han as reference populations. The Lebanese Christians (A) do not appear admixed from these sources but the Lebanese Muslims (B) similarly to the Turkish (C) have weighted LD curves displaying a clear decay and signaling admixture.

Table S1. Information about radiocarbon calibration

		Calibration data set: intcal13.14c[a]		
**ID**	**Radiocarbon Age BP**	**% area enclosed**	**cal AD age ranges**	**relative area under probability distribution**
**SI-39**	780 +/- 35	68.3 (1 sigma)[b]	cal AD 1224- 1237	0.303
			1241- 1269	0.697
		95.4 (2 sigma)[c]	cal AD 1191- 1283	1
**SI-41**	812 +/- 22	68.3 (1 sigma)	cal AD 1218- 1254	1
		95.4 (2 sigma)	cal AD 1187- 1266	1
**SI-45**	774 +/- 27	68.3 (1 sigma)	cal AD 1225- 1232	0.158
			1244- 1272	0.842
		95.4 (2 sigma)	cal AD 1219- 1278	1
**SI-42**	811 +/- 45	68.3 (1 sigma)	cal AD 1190- 1199	0.082
			1203- 1265	0.918
		95.4 (2 sigma)	cal AD 1058- 1075	0.016
			1154- 1281	0.984
**SI-53**	949 +/- 29	68.3 (1 sigma)	cal AD 1029- 1050	0.253
			1083- 1126	0.562
			1135- 1151	0.185
		95.4 (2 sigma)	cal AD 1025- 1154	1
**QED-2**	1712 +/- 36	68.3 (1 sigma)	cal AD 258- 283	0.273
			322- 387	0.727
		95.4 (2 sigma)	cal AD 244- 400	1
**QED-4**	1517 +/- 49	68.3 (1 sigma)	cal AD 433- 459	0.186
			466- 489	0.159
			532- 603	0.655
		95.4 (2 sigma)	cal AD 426- 632	1
**QED-7**	1734 +/- 33	68.3 (1 sigma)	cal AD 251- 343	1
		95.4 (2 sigma)	cal AD 237- 389	1

a Reimer et al. 2013
b square root of (sample std. dev.^2 + curve std. dev.^2)
c 2 x square root of (sample std. dev.^2 + curve std. dev.^2)

Table S2. Contamination estimates from mtDNA and male X chromosome analysis.

ID	mtDNA contamination %	Genomic contamination %
SI-38	0-2	0.4-0.7
SI-39	0-2	0.5-1.6
SI-40	0-2	1.5-1.6
SI-41	0-2	0.6-0.8
SI-42	0-2	0.5-1
SI-44	0-2	0-1.7
SI-45	0-2	0.6-.08
SI-47	0-2	0-1.4
SI-53	0-2	0.4-0.5
QED-2	0-2	0.2-0.4
QED-4	0-2	-
QED-7	0-2	-
QED-9	8-10	-
QED-12	0-2	-

Table S3. Modeling SI-41 as a mixture of a European population *A* and a Near Eastern population *B*. Using *qpAdm*, we set the outgroups as Ust'-Ishim, Kostenki14, MA1, Onge, Papuans, Chukchi, Karitiana, Eastern hunter-gatherers (EHG), Western hunter-gatherers (WHG), Natufians, Caucasus hunter-gatherers (CHG), Neolithic Iranians, Neolithic Anatolians, and Neolithic Levantines and tested if SI-41 can be modelled as being descendent from a mixture of European and Near Eastern ancestries. We report the top 20 models based on their P-value for the rank=1 matrix and highlight in red the instances where the P-value is >0.05 indicating the model cannot be rejected.

			Mixture proportions		
**A**	**B**	**P value for rank=1**	**A**	**B**	**Std. Error**
Spanish_North	Saudi	2.99E-01	0.538	0.462	0.054
Basque	Saudi	2.33E-01	0.536	0.464	0.053
Spanish_North	BedouinB	1.93E-01	0.57	0.43	0.052
Basque	BedouinB	1.55E-01	0.565	0.435	0.05
Spanish	Saudi	1.12E-01	0.623	0.377	0.063
Spanish	BedouinB	9.42E-02	0.651	0.349	0.059
Spanish_North	BedouinA	4.47E-02	0.577	0.423	0.053
Basque	BedouinA	3.46E-02	0.574	0.426	0.051
Spanish_North	Palestinian	2.10E-02	0.557	0.443	0.057
Spanish_North	Jew_Turkish	1.72E-02	0.383	0.617	0.081
Spanish	BedouinA	1.72E-02	0.671	0.329	0.061
Basque	Palestinian	1.49E-02	0.553	0.447	0.055
Basque	Jew_Turkish	1.49E-02	0.378	0.622	0.08
Spanish_North	Lebanon_MP	1.06E-02	0.528	0.472	0.064
Spanish	Jew_Turkish	9.61E-03	0.477	0.523	0.104
Spanish	Palestinian	9.18E-03	0.656	0.344	0.066
Basque	Lebanon_MP	6.68E-03	0.526	0.474	0.062
Spanish	Lebanon_MP	6.53E-03	0.633	0.367	0.076
Italian_North	Saudi	5.45E-03	0.668	0.332	0.072
Italian_North	BedouinB	4.90E-03	0.697	0.303	0.066

Table S4. Modeling SI-53 as a mixture of a European population *A* and a Near Eastern population *B*. We used *qpAdm* as described in Table S3 to test individual SI-53.

			Mixture proportions		
**A**	**B**	**P value for rank=1**	**A**	**B**	**Std. Error**
Croatian	Lebanon_MP	8.92E-01	0.483	0.517	0.063
Norwegian	Lebanon_MP	8.48E-01	0.365	0.635	0.048
Croatian	Jew_iraqi	8.20E-01	0.498	0.502	0.06
Romanian	Lebanon_MP	8.16E-01	0.532	0.468	0.07
Croatian	Lebanese_Christian	8.06E-01	0.464	0.536	0.064
Spanish_North	Assyrian	8.04E-01	0.43	0.57	0.053
Italian_North	Assyrian	7.99E-01	0.603	0.397	0.074
Romanian	Jew_iraqi	7.94E-01	0.549	0.451	0.066
Spanish	Assyrian	7.88E-01	0.512	0.488	0.063
Hungarian	Lebanon_MP	7.65E-01	0.422	0.578	0.057
German	Lebanon_MP	7.52E-01	0.39	0.61	0.053
Basque	Assyrian	7.35E-01	0.425	0.575	0.052
Italian_North	Syrian	7.29E-01	0.591	0.409	0.077
Romanian	Lebanese_Christian	7.26E-01	0.515	0.485	0.071
English	Lebanon_MP	7.26E-01	0.4	0.6	0.054
French	Jew_iraqi	7.26E-01	0.451	0.549	0.055
Spanish	Jew_iraqi	6.92E-01	0.517	0.483	0.063
Croatian	Jew_Turkish	6.86E-01	0.324	0.676	0.084
French	Lebanon_MP	6.83E-01	0.431	0.569	0.059
Spanish_North	Jew_iraqi	6.55E-01	0.433	0.567	0.054

Table S5. Modeling the Crusaders' pit Europeans similarly to SI-41 (Table S3) and SI-53 (Table S4). We used *qpAdm* as described in Table S3 and show the top 8 models for *Test* based on their P-value for the rank=1 matrix.

				Mixture proportions		
**Test**	**A**	**B**	**P value for rank=1**	**A**	**B**	**Std. Error**
SI-39	English	Lebanon_MP	5.45E-01	0.94	0.06	0.059
SI-39	English	Jew_Iraqi	5.24E-01	0.948	0.05	0.056
SI-39	English	Lebanese_Christian	5.09E-01	0.95	0.05	0.06
SI-39	German	Lebanon_MP	4.99E-01	0.912	0.09	0.057
SI-39	English	Jew_Turkish	4.96E-01	0.947	0.05	0.073
SI-39	English	Assyrian	4.91E-01	0.959	0.04	0.06
SI-39	English	Saudi	4.88E-01	0.963	0.04	0.054
SI-39	English	Palestinian	4.84E-01	0.962	0.04	0.058
SI-40	Basque	Lebanese_Christian	9.61E-01	0.835	0.17	0.066
SI-40	Basque	Assyrian	9.56E-01	0.851	0.15	0.06
SI-40	Basque	BedouinB	9.55E-01	0.856	0.14	0.058
SI-40	Basque	Lebanon_MP	9.55E-01	0.841	0.16	0.065
SI-40	Basque	Syrian	9.52E-01	0.842	0.16	0.064
SI-40	Basque	Jew_Turkish	9.51E-01	0.793	0.21	0.084
SI-40	Basque	Saudi	9.45E-01	0.854	0.15	0.06
SI-40	Basque	Jew_iraqi	9.37E-01	0.857	0.14	0.06
SI-47	German	BedouinB	5.87E-01	0.93	0.07	0.056
SI-47	German	Jew_Turkish	5.63E-01	0.914	0.09	0.075
SI-47	German	Lebanon_MP	5.55E-01	0.931	0.07	0.063
SI-47	German	Saudi	5.52E-01	0.937	0.06	0.057
SI-47	German	BedouinA	5.36E-01	0.939	0.06	0.06
SI-47	German	Lebanese_Christian	5.24E-01	0.941	0.06	0.064
SI-47	German	Palestinian	5.24E-01	0.942	0.06	0.062
SI-47	Croatian	Assyrian	5.23E-01	1.185	-0.19	0.081

Table S6. Modeling Ashkenazi Jews, Sicilians and Southern Italians similarly to SI-41 (Table S3) and SI-53 (Table S4). We used *qpAdm* as described in Table S3 and show the top 8 models for *Test* based on their P-value for the rank=1 matrix.

				**Mixture proportions**		
**Test**	**A**	**B**	**P value for rank=1**	**A**	**B**	**Std. Error**
Ashkenazi Jew	Hungarian	Jew_Turkish	4.94E-01	0.199	0.801	0.028
Ashkenazi Jew	English	Jew_Turkish	4.89E-01	0.187	0.813	0.026
Ashkenazi Jew	Norwegian	Jew_Turkish	4.87E-01	0.163	0.837	0.023
Ashkenazi Jew	Croatian	Jew_Turkish	4.58E-01	0.23	0.77	0.032
Ashkenazi Jew	German	Jew_Turkish	4.17E-01	0.179	0.821	0.026
Ashkenazi Jew	French	Jew_Turkish	3.60E-01	0.207	0.793	0.029
Ashkenazi Jew	Romanian	Jew_Turkish	3.50E-01	0.269	0.731	0.038
Ashkenazi Jew	Spanish	Lebanese_Christian	2.60E-01	0.441	0.559	0.026
Sicilian	Italian_North	Jew_Turkish	8.10E-01	0.401	0.599	0.043
Sicilian	Spanish	Jew_Turkish	8.00E-01	0.302	0.698	0.033
Sicilian	Basque	Jew_Turkish	7.29E-01	0.232	0.768	0.027
Sicilian	Spanish_North	Jew_Turkish	7.23E-01	0.233	0.767	0.027
Sicilian	Italian_South	Lebanon_MP	6.23E-01	1.186	-0.186	0.105
Sicilian	French	Jew_Turkish	5.60E-01	0.221	0.779	0.026
Sicilian	Italian_South	Jew_iraqi	4.49E-01	1.121	-0.121	0.072
Sicilian	Italian_South	Lebanese_Christian	4.45E-01	1.155	-0.155	0.096
Italian_South	Italian_North	Lebanon_MP	5.13E-01	0.512	0.488	0.044
Italian_South	Italian_North	Jew_Turkish	3.90E-01	0.266	0.734	0.064
Italian_South	Spanish	Lebanon_MP	3.52E-01	0.406	0.594	0.038
Italian_South	Romanian	Lebanon_MP	3.26E-01	0.407	0.593	0.039
Italian_South	French	Lebanon_MP	2.43E-01	0.329	0.671	0.033
Italian_South	Italian_North	Lebanese_Christian	2.26E-01	0.512	0.488	0.041
Italian_South	Spanish_North	Lebanon_MP	2.24E-01	0.338	0.662	0.035
Italian_South	Basque	Lebanon_MP	2.23E-01	0.334	0.666	0.033
